# Multispecies Biofilms Treated With Endodontic Sealers or Calcium Hydroxide: Antimicrobial Activity and Changes in Community Composition

**DOI:** 10.1111/iej.70015

**Published:** 2025-08-13

**Authors:** Steven K. Uttech, Ronald Ordinola‐Zapata, W. Craig Noblett, Maria Martell, Bruno Lima, Christopher Staley

**Affiliations:** ^1^ Division of Endodontics, Department of Restorative Sciences, School of Dentistry University of Minnesota Minneapolis Minnesota USA; ^2^ Division of Basic & Translational Research, Department of Surgery University of Minnesota Minneapolis Minnesota USA; ^3^ Division of Basic Sciences, Department of Diagnostic and Biological Sciences, School of Dentistry University of Minnesota Minneapolis Minnesota USA

**Keywords:** endodontic sealers, intracanal medication, microbiome, next generation sequencing

## Abstract

**Introduction:**

To evaluate the antimicrobial activity and changes in community composition in biofilms treated with two root canal sealers or calcium hydroxide.

**Material and Methods:**

Forty‐nine extracted bovine dentine cylinders were inoculated with subgingival dental plaque for 2 weeks in a CDC biofilm reactor. Three treatment groups were assigned: AH Plus, BC Sealer, and calcium hydroxide. Propylene glycol inert vehicle (PG) and untreated contaminated samples were used as controls. The infected root canal space was in direct contact with the materials for 7 days under anaerobic incubation. Anaerobic culture (colony forming units, CFU), quantification PCR (qPCR), and next generation sequencing were used to assess the effect of each material. Differences in the number of molecules (qPCR), CFU, and abundances of genera were evaluated using the Kruskal–Wallis test. The Shannon and Chao1 indices were used to measure alpha diversity. Differences in community composition were evaluated using non‐parametric analysis of similarity (ANOSIM).

**Results:**

The CFU, Shannon, and Chao1 indices revealed significant differences between BC Sealer, AH Plus, and calcium hydroxide groups versus the untreated control group and PG (*p* < 0.003). Calcium hydroxide and BC Sealer significantly reduced the qPCR values compared to the control group and PG. The biofilm composition (98.5%) was primarily composed of *Peptostreptococcus*, *Streptococcus*, *Parvimonas*, *Fusobacterium*, *Veillonela*, *Mogibacterium*, *Lancefieldella*, *Eubacterium*, *Slackia*, and *Prevotella*. Significant differences in overall community composition and beta diversity between untreated controls and AH Plus were observed (ANOSIM *R* = 0.674, *p* < 0.001). *Parvimonas*, *Streptococcus*, *Eubacterium*, and *Lancefieldella* were not affected by any of the materials tested.

**Conclusion:**

Calcium hydroxide and BC Sealer significantly reduced the viability and the total number of DNA copies. AH Plus sealer reduced the bacterial viability but did not affect the DNA concentration. AH Plus significantly alters overall biofilm community composition compared to other groups. None of the materials tested eliminated the multispecies biofilm completely.

## 1 Introduction

Endodontic treatment is based on the biological principles of debridement, disinfection, and obturation, creating an environment conducive to repair and healing (Schilder 1974). In the root canal filling phase, a root canal sealer is employed in conjunction with gutta‐percha. Among the available materials, calcium silicate‐based bioceramics have gained relevance and popularity in both surgical and non‐surgical endodontic procedures (Wang et al. 2021).

Calcium hydroxide is a common component in calcium silicate‐based bioceramic sealers (Lim et al. 2020). Early studies revealed that first‐generation calcium silicate materials, such as mineral trioxide aggregate (MTA), formed a bacterial‐resistant barrier, a property attributed to the calcium hydroxide byproduct generated during hydration (Torabinejad et al. 1995; Farrugia, Baca, et al. 2017; Camilleri et al. 2022). Similarly, the antibacterial mechanism of calcium silicate‐based bioceramic sealers is explained by their ability to create an alkaline environment through the release of hydroxyl ions (Lim et al. 2020; Farrugia, Haider, et al. 2017; Bose et al. 2020).

Multiple methods have been used to assess the antimicrobial activity of different bioceramic sealers, resulting in conflicting results. Prior researchers have employed *Enterococcus faecalis* biofilm models to explore the antimicrobial properties of different sealers (Wang et al. 2014, 2021; Chen et al. 2024). Other authors (Kapralos et al. 2018) have challenged different sealers against 24‐h single‐species biofilms of *E. faecalis* , *Staphylococcus epidermidis* , *Staphylococcus aureus* , and *Streptococcus mutans* . Their findings revealed that bacteria displayed higher susceptibility to epoxy resin‐based sealers in comparison to bioceramics.

Bose et al. (2020) employed confocal laser scanning microscopy (CLSM) and a culture‐based methodology to evaluate the impact of bioceramic, zinc oxide‐eugenol and epoxy resin sealers on biofilms formed by *Actinomyces radicidentis* , *S. epidermidis* , *Streptococcus oralis* and *E. faecalis* using a direct contact test. Although bioceramic sealers demonstrated enhanced anti‐biofilm activity relative to epoxy‐resin based sealers, biofilm inhibition assays indicated that viable bacterial counts remained consistently high, ranging from 9 to 10 log_10_ colony forming units (CFU) for all tested sealers and controls.

Prior authors have recognised the limitations of using planktonic bacteria, single‐species models or immature biofilms (24 h) to assess the antimicrobial efficacy of biomaterials in endodontics. These approaches do not accurately reflect the complexity of biofilms found in the root canal system (Pappen et al. 2010; Stojicic et al. 2012; Kreth et al. 2019; Bose et al. 2020). Given the well‐documented role of anaerobic bacteria in endodontic failures, this study aims to overcome previous limitations by employing a multispecies biofilm in an accepted model to study dentinal infection (Haapasalo and Ørstavik 1987; Siqueira and de Uzeda 1996; Saleh et al. 2004). In order to provide a better understanding of the interactions between endodontic materials and multispecies biofilms, this study will evaluate two root canal sealers (AH Plus and BC Sealer) in conjunction with an intracanal medication (calcium hydroxide) under similar experimental conditions. The central hypothesis of this study is that calcium hydroxide (Ultracal, South Jordan, UT, USA), BC Sealer (Brasseler, Savannah, GA, USA) and AH Plus (Dentsply Sirona, Charlotte, NC, USA) will demonstrate superior antimicrobial activity compared to an inert compound (propylene glycol) under standardised experimental conditions.

## 2 Materials and Methods

This study tested the antimicrobial properties of two root canal sealers: AH Plus (Dentsply Sirona, Charlotte, NC, USA), BC Sealer (Brasseler, Savannah, GA, USA) and one intracanal medicament, calcium hydroxide (Ultracal, South Jordan, UT, USA). Propylene glycol (vehicle) and untreated contaminated samples were used as controls. The institutional IRB protocol stated that the model used in this project is not considered human research (Protocol Study 0014237, 0024340). The PRILE flowchart can be found in Figure S1.

Forty‐nine extracted, intact bovine incisors were used. The apical 5 mm and the crown 1 mm below the CEJ were removed with a rotating diamond saw following the methodology of Haapasalo and Ørstavik (1987). Canals were widened to 1.4 mm in diameter using a reamer bur size 4. This left the cylinders with a dimension of approximately 10 mm in height and 6 mm in diameter. The cylinders were autoclaved at 121°C for 15 min. This method of preparing dentine cylinders was previously described by Haapasalo and Ørstavik (1987).

### 2.1 Power Analysis

Pilot data using CFU and Quantification PCR (qPCR) data from three samples per group revealed that the effect size of the experiment was 1.4. Thus, a minimum of three samples per group was required to detect differences in culture and qPCR levels (0.8 power, *α* = 0.05). Data were calculated in G* power software. For sequencing analysis, even 5 samples allow detection of an effect size (*ω*2) 0.3 with a power of 0.90 and *α* = 0.05 (Kelly et al. 2015).

### 2.2 Root Canal Contamination

Root canal specimens that were prepared underwent inoculation with 100 μL of subgingival human‐derived dental plaque, obtained from a single donor and previously diluted in anaerobic transport medium (Anaerobe System, Morgan Hill, CA, USA). The Center for Disease Control (CDC) Biofilm Reactor (BioSurface Technologies, Bozeman, MT, USA) underwent an initial 24‐h incubation with the subgingival plaque under shear conditions, without media flow. Following the incubation, Columbia medium (BD Difco, Detroit, MI, USA) was pumped through the reactor at a flow rate of 2.5 L/24 h for a duration of 14 days (Rudney et al. 2012; Coaguila‐Llerena et al. 2022). The internal temperature of the reactor vessel was maintained at 37°C, and the stirring rate was set to 90 rpm. The protocol for the establishment of this biofilm model was previously reported (Rudney et al. 2012). Subsequently, the samples were retrieved from the vessel utilising sterile instruments for further treatment. See Figure S2.

### 2.3 Root Canal Preparation and Microbiological Sampling

To ensure reliable results, 10–12 samples per group were used to test the antimicrobial efficacy of the materials tested; calcium hydroxide (Ultracal, Ultradent, South Jordan, UT, USA), AH Plus (Dentsply, Konstanz, Germany) and BC Sealer (Brasseler USA). Five samples were used as fresh controls (no treatment after 14 days in the biofilm reactor), and 10 samples were treated with propylene glycol–inert vehicle control. Two additional samples were used for SEM imaging and microscopic biofilm confirmation. The sealers, calcium hydroxide paste and propylene glycol were placed individually on the bottom of the wells to cover the floor of the well (24 well cell culture plates). The dentine cylinders were rinsed previously with sterile phosphate‐buffered saline (PBS) and then placed in the well using a sterile instrument. The root canal space was filled with the selected materials using a syringe. The canal space was overfilled, allowing treatment material to cover the exposed coronal surface of the dentine cylinder. The culture plates with the specimens were then stored in an anaerobic chamber (10% H_2_, 10% CO_2_, and 80% N_2_) at 37°C for 7 days.

After the incubation period, samples were then removed from the anaerobic chamber and a sterile #4 round bur was utilised to remove set treatment material from the canal space without disrupting dentine. Dentine chips from the root canal were collected using a sterilized #6 Gates Glidden drill using low speed for 5 s; the chips were collected in a tube prepared with sterile PBS.

### 2.4 Culture Analysis, DNA Extraction and Sequencing Analysis

Serial dilutions of each microbiological sample were prepared and 100 μL were plated on Columbia agar plates (BD Difco) containing hemin 0.5 mg/mL + menadione 5 mg/mL. The plates were incubated under anaerobic conditions for 48 h. CFU were quantified, and the results were expressed using the logarithmic scale.

### 2.5 DNA Extraction and Sequencing

Bacterial DNA from biological samples was extracted using the DNeasy PowerLyzer PowerSoil Kit (Qiagen, Hilden, Germany). PCR quantification and sequencing were performed by the University of Minnesota Genomics Center (Gohl et al. 2016). The V3‐V4 hypervariable region of the 16S rRNA gene was amplified and paired‐end sequenced using the S‐D‐Bact‐0341‐b‐S‐17/S‐D‐Bact‐0785‐a‐A‐21 primer set (Klindworth et al. 2013) on the Illumina MiSeq Platform (Illumina Inc., San Diego, CA, USA) at a read length of 301 nucleotides (nt) (Gohl et al. 2016; Caporaso et al. 2012). Raw data are deposited in the Sequence Read Archive under accession number SRP499529.

### 2.6 16S rRNA Processing and Analysis

All sequence processing was performed using Mothur software (version 1.41.1), as previously described (Schloss et al. 2009; Staley et al. 2017; Park et al. 2024). Sequences were pair‐end‐joined and trimmed for quality and aligned against the SILVA database (version 138.1) for clustering. Sequencing errors were removed using a 2% pre‐clustering step (Huse et al. 2010); chimeras were removed using UCHIME (version 4.2.40) (Edgar et al. 2011). Operational taxonomic units (OTUs) were binned at 99% similarity using the OptiClust algorithm (Westcott and Schloss 2017). OTUs were classified against the Ribosomal Database Project release (version 18). Different databases were used for alignment and classification because of processing considerations described previously (Schloss et al. 2011). The Shannon and Weaver (1949) and Chao (1984) indices were calculated in Mothur and used to measure alpha (within‐sample) diversity. The Bray and Curtis (1957) dissimilarity index was used to measure beta (between‐sample) diversity and was visualised by ordination using principal coordinate analysis (Anderson and Willis 2003).

### 2.7 Statistical Analysis

Differences in CFU, qPCR and abundances of genera were evaluated using the Kruskal–Wallis test. Differences in alpha diversity were compared using ANOVA with Tukey's post hoc test using XLSTAT software (version 2023.3.1.1416; Addinsoft; Belmont, MA, USA). A covariance matrix correction to meet the assumptions of heteroscedasticity was performed. Furthermore, the least squares mean was used to account for unbalanced data. Differences in community composition were evaluated using non‐parametric analysis of similarity (ANOSIM) in Mothur (Clarke 1993). All statistical analyses were performed at *ɑ* = 0.05, and ANOSIM analyses were Bonferroni‐corrected for pairwise comparisons.

## 3 Results

### 3.1 SEM Images

**FIGURE 1:**
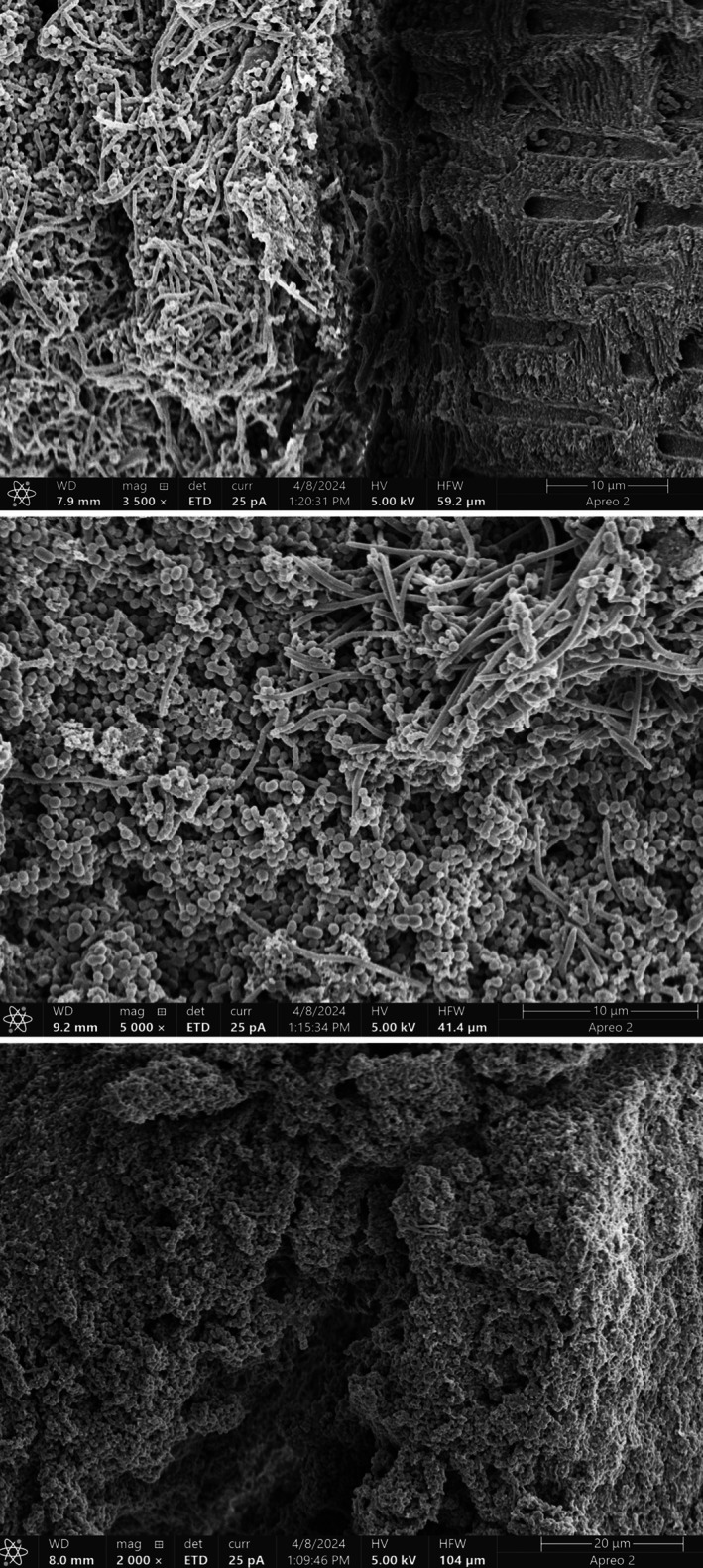
SEM images of biofilms of the immediate control. Images on top and centre were obtained at 3500 and 5000× magnification, and the image at the bottom is at 2000× magnification.

Densely packed bacterial cells were observed adhering to the dentine surface, forming a confluent biofilm layer. The biofilm appeared highly heterogeneous, with varying morphologies and arrangements of microbial cells. The intimate association between the bacterial biofilm and the dentinal surface was evident, with the bacterial cells appearing to colonise not only the surface but also the exposed dentinal tubules. See Figure 1.

### 3.2 CFU

**FIGURE 2:**
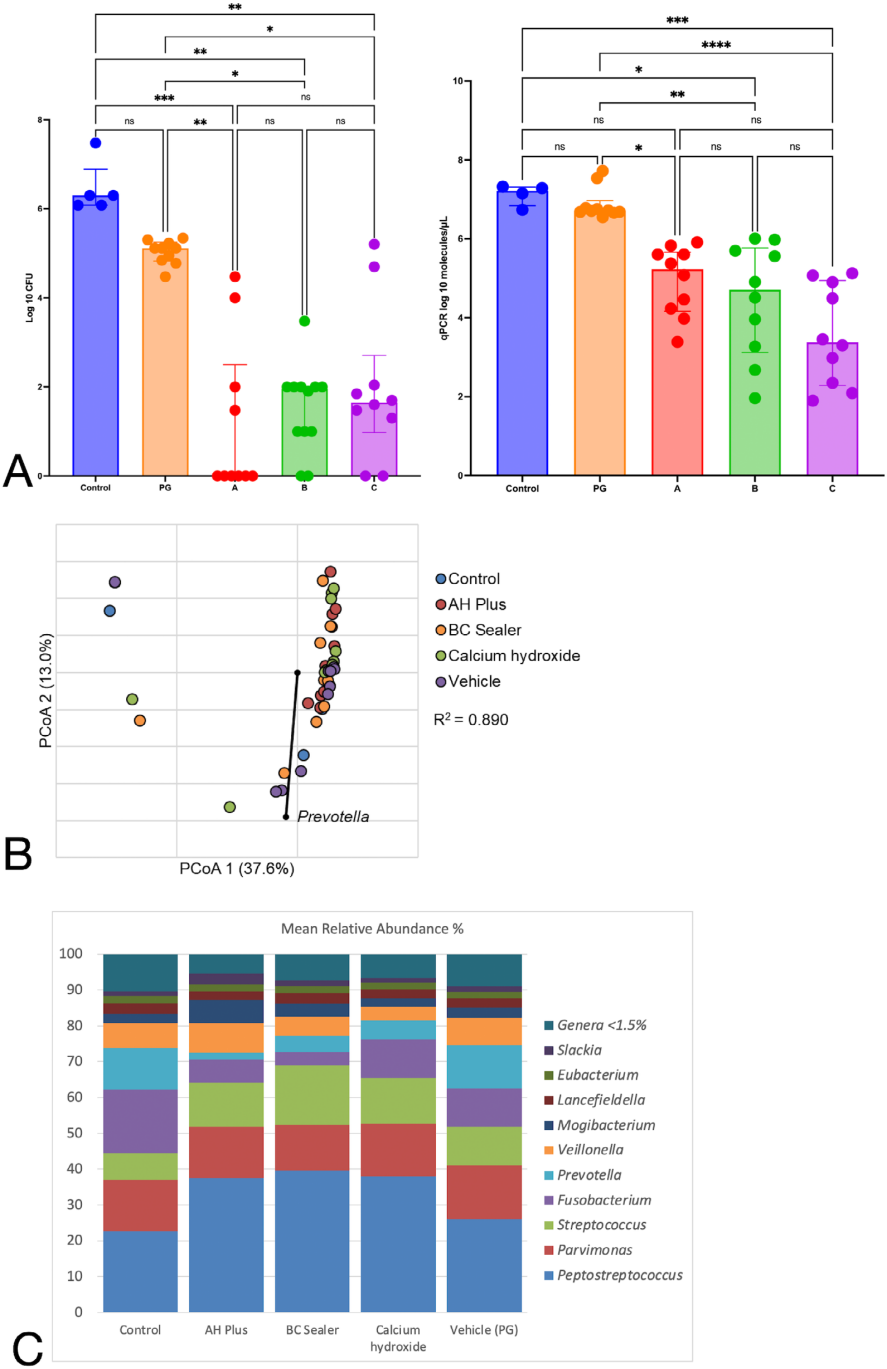
(A) Kruskal–Wallis analysis comparing culture and qPCR results among the treatment and control groups on a log_10_ scale. Statistically significant differences are noted by the asterisks. PG (Inert vehicle), A (AH Plus), B (BC Sealer), C (Calcium Hydroxide), Control (untreated). (B) Principal coordinate analysis of Bray–Curtis dissimilarity matrices. Differences between untreated controls and AH Plus (ANOSIM *R* = 0.674, *p* < 0.001, Bonferroni‐corrected *ɑ* = 0.003). Vehicle (PG), control (untreated) (C). Distribution of abundant genera among groups. Genera with a mean abundance < 1.5% among all groups were consolidated. Vehicle = propylene glycol.

The culture analysis showed that AH Plus, BC Sealer and calcium hydroxide promoted log_10_ median and range values of 0 (0–4.47), 1.95 (0–3.47) and 1.65 (0–5.20) respectively, whereas propylene glycol and the untreated control group demonstrated log_10_ values of 5.11 (4.48–5.34) and 6.30 (6.07–7.46), respectively. AH Plus, BC Sealer and calcium hydroxide all demonstrated lower CFU values when compared to the untreated control group and propylene glycol (*p* < 0.001). No significant difference between both sealers and calcium hydroxide groups (*p* > 0.999), as well as between the control and propylene glycol groups, was observed (*p* > 0.999). See Figure 2.

### 3.3 qPCR

Two samples from the BC Sealer group and one from the untreated control group were lost during DNA extraction. The results from qPCR are shown in Figure 2. The qPCR analysis showed that AH Plus, BC Sealer and calcium hydroxide promoted log_10_ median and range values of 5.22 (3.38–5.91), 4.70 (1.95–6.00) and 3.37 (1.89–5.12), respectively, whereas propylene glycol and the control group demonstrated log_10_ values of 6.71 (6.55–7.72) and 7.21 (6.73–7.32), respectively. Calcium hydroxide significantly reduced the number of qPCR molecules compared to the control and propylene glycol groups (*p* < 0.0001, *p* = 0.0008, respectively). The same results were noted for BC Sealer (*p* = 0.021, *p* < 0.0001). No significant differences were observed between AH Plus, BC Sealer and calcium hydroxide groups (*p* > 0.999) and between AH Plus and control (*p* = 0.067). No significant differences between control and propylene glycol were observed (*p* > 0.999).

### 3.4 Next‐Generation Sequencing (NGS)

Sequencing analysis showed that the number of operational taxonomic units in the untreated control group was 231.5 + 104. The number of reads, OTUs, Shannon Index and Chao1 Index can be found in Table 1. There was no significant difference in the number of reads among groups (*p* = 0.228). AH Plus, BC Sealer and calcium hydroxide significantly reduced the number of observed species (sobs‐OTUs), as well as the biofilm species richness and evenness. All data presented in Table 1 had a normal distribution except for Shannon alpha diversity values; the same test was repeated using a non‐parametric Kruskal–Wallis test with false discovery rate corrections. The *p*‐value and multiple comparisons did not differ from the data obtained with ANOVA. Figure 2 shows the community composition of the immediate control group and the effect on the composition following each treatment.

**TABLE 1:** Mean and standard deviation of sequencing data from all samples including OTUs, Shannon and Chao1 alpha diversity indices.

Treatment	Reads	Coverage	OTUs	Shannon	Chao1
Control (fresh)	34 798 ± 23 408	98.8 ± 1.6^B^	231.5 ± 104.7^A^	2.74 ± 0.14^A^	599 ± 251^A^
AH Plus	50 658 ± 15 953	99.8 ± 0.1^A^	184.3 ± 47.0^B^	2.23 ± 0.24^B^	429 ± 168^B^
BC Sealer	34 751 ± 21 653	99.7 ± 0.2^A^	144.3 ± 64.7^B^	2.25 ± 0.37^B^	320 ± 209^B^
Calcium hydroxide	54 265 ± 29 385	99.8 ± 0.1^A^	173.2 ± 74.5^B^	2.26 ± 0.27^B^	351 ± 209^B^
Inert vehicle (PG)	38 898 ± 19 820	99.3 ± 0.7^AB^	291.1 ± 113.6^A^	2.75 ± 0.11^A^	772 ± 308^A^
*p*‐value	0.228	0.024	0.003	< 0.0001	0.0003

*Note:* Different uppercase letters in each column indicate significant differences by Tukey's post hoc test (*p* < 0.05).

The main dominating biofilm taxa identified across all samples (98.5%) were primarily categorised into 10 microbial genera, including *Peptostreptococcus*, *Streptococcus*, *Parvimonas*, *Fusobacterium*, *Veillonella*, *Mogibacterium*, *Lancefieldella*, *Eubacterium*, *Slackia and Prevotella*. Regarding relative abundance, minimal changes after treatment compared to the untreated control or the inert vehicle were observed for *Fusobacterium*, *Prevotella*, *Veillonella*, *Peptostreptococcus*, *Slackia and Mogibacterium*. The exception was BC Sealer, which significantly reduced the relative abundance of *Fusobacterium*. AH Plus significantly reduced the relative abundance of *Prevotella* (See Table 2). The relative abundance of *Parvimonas*, *Streptococcus*, *Eubacterium* and *Lancefieldella* was not affected by any of the materials tested.

**TABLE 2:** Relative abundances of bacterial taxa among biofilms from treatment groups compared to untreated control via Kruskal–Wallis test.

Treatment	*Fusobacterium*	*Prevotella*	*Veillonella*	*Mogibacterium*	*Slackia*
Control (fresh)	17.7 ± 3.2^A^	11.6 ± 4.0^AB^	7.0 ± 1.6^AB^	2.4 ± 0.5^B^	1.4 ± 0.6^B^
AH Plus	6.5 ± 3.6^bc^	1.8 ± 1.6^C^	8.3 ± 3.7^A^	6.4 ± 2.6^A^	3.0 ± 1.2^A^
BC Sealer	3.7 ± 2.1^C^	4.7 ± 3.3^bc^	5.4 ± 3.3^AB^	3.6 ± 1.2^AB^	1.6 ± 0.6^B^
Calcium hydroxide	10.9 ± 7.3^ABC^	5.2 ± 3.2^B^	3.9 ± 1.1^B^	2.3 ± 0.6^B^	1.3 ± 0.9^B^
Inert Vehicle (PG)	10.6 ± 4.2^AB^	12.1 ± 2.9^A^	7.6 ± 2.3^A^	2.9 ± 1.1^B^	1.5 ± 0.6^B^
*p*‐value	< 0.0004	< 0.0001	0.009	< 0.0003	0.003

*Note:* Different uppercase letters in each column indicate significant differences (*p* < 0.05).

Significant differences in overall community composition and beta diversity between AH Plus and untreated controls or the inert control (PG) were observed (ANOSIM *R* = 0.674, *p* < 0.001, *R* = 0.218, *p* < 0.001; Bonferroni‐corrected *ɑ* = 0.003). No significant difference in beta diversity between untreated controls and all other groups was noted (ANOSIM *R* = 0.171–0.188, *p* = 0.148–0.172, Bonferroni‐corrected *ɑ* = 0.005). Figure 2.

## 4 Discussion

The hypothesis that BC Sealer, AH Plus and calcium hydroxide will exhibit substantial antimicrobial activity compared to the inert control was accepted. It is known that bacteria in the root canal system can establish complex biofilms comprised of multiple anaerobic species that can be challenging to eliminate (Ricucci and Siqueira 2010; Ordinola‐Zapata et al. 2024). In this study, the multispecies biofilm composition included microorganisms implicated in primary and secondary endodontic infections, including *Fusobacterium*, *Parvimonas*, *Peptostreptococcus*, *Streptococcus* and *Prevotella* (Ordinola‐Zapata et al. 2023, 2024). The biofilm reactor was able to produce microbial communities that exhibited tolerance to calcium hydroxide and endodontic sealers. Because intracanal medication and sealers have different but complementary functions in endodontic treatment, their inclusion was justified. In the present study, calcium hydroxide was included as a reference because of its longstanding use as an intracanal medicament and its known antimicrobial effect after at least 1 week of intracanal application (Sjogren et al. 1991). Although the antimicrobial properties of endodontic sealers are often considered secondary to their sealing ability, dimensional stability and biocompatibility, many commercially available root canal sealers exhibit varying degrees of antimicrobial activity (Saavedra et al. 2022; Liu et al. 2025). Assessing these effects in vitro provides insights into their potential to affect established mature root canal biofilms.

Although a baseline quantification of bacterial or DNA load prior to treatment was not feasible without disrupting the biofilm in the dentine block model, this study employed a standardised biofilm cultivation protocol using a CDC reactor and included both ‘fresh’ and ‘vehicle’ controls to approximate pre‐operative microbial conditions. These controls allowed us to understand the relative efficacy of each sealer while preserving the biofilm's structural architecture for post‐treatment analysis. From the methodological point of view, this study used a multimethod approach that included culture and molecular techniques. Although culture‐based methods are inherently biased toward fast‐growing microorganisms (Gupta et al. 2019; Siqueira and Rôças 2022), the multimethod approach—combining CFU, qPCR and next‐generation sequencing—allowed us to obtain a comprehensive assessment of both microbial viability and taxonomic shifts. Similarly, despite the well‐known limitations of DNA‐based approaches, a recent study (Nogales et al. 2025) found that DNA‐positive samples also tested positive for rRNA, indicating that the likelihood of false‐positive results from DNA‐based datasets (qPCR) is minimal.

In the first part of the study, the culture technique (CFU) data indicated lower values for calcium hydroxide and the bioceramic sealer in comparison to the inert control (6.30 log_10_). The antimicrobial property of bioceramic sealers is attributed to the hydration reaction, resulting in the formation of calcium silicate hydrogel and calcium hydroxide (Zhou et al. 2013; Wang et al. 2014; Camilleri et al. 2022). These compounds are responsible for elevating a high pH level within the root canal system. Hydroxyl ions promote an alkaline environment, damaging cell membranes and producing protein denaturation (Siqueira and Lopes 1999; Saavedra et al. 2022). Although BC Sealer revealed a reduction in the relative abundance of *Fusobacterium* and *Prevotella*, the effect of calcium hydroxide and BC Sealer appeared to be quantitative and did not impact the overall biofilm community composition, as revealed by the beta diversity analysis. Multiple studies have highlighted the limited efficacy of calcium hydroxide in eliminating bacterial cells within dentinal tubules. Haapasalo and Ørstavik (1987) observed that a calcium hydroxide paste (Calasept, Swedia, Knivsta, Sweden) failed to eliminate *E. faecalis* . Safavi et al. (1990) demonstrated the persistence of viable *E. faecalis* in dentinal tubules following prolonged treatment with calcium hydroxide. This limited effect could be explained by the buffering capacity of dentine (Farrugia, Baca, et al. 2017). Another explanation is the general agreement that biofilm cells can possess a superior capacity for survival at alkaline pH (Chávez de Paz et al. 2007). In addition, many anaerobic isolates can withstand alkaline stress up to a pH of 9 (Lew et al. 2015), including *Enterococcus*, *Fusobacterium*, *Parvimonas* and *Peptostreptococcus*. In vitro data have also revealed that biofilms of *Streptococcus anginosus* , *Streptococcus gordonii* , and *S. oralis* can survive alkaline environments of pH 10.5 (Chávez de Paz et al. 2007, 2010). The microbial characterisation of the post‐treatment samples confirmed that most of the residual microorganisms were anaerobic or facultative. In this study, the gram‐positive *Parvimonas*, *Streptococcus*, *Eubacterium* and *Lancefieldella* were not affected by any of the materials tested. Multiple factors can affect microbial resistance, including natural resistance mechanisms (Chávez de Paz et al. 2007), the presence of organic components (Portenier et al. 2001) and the ability of microorganisms to penetrate deep into dentinal tubules, avoiding exposure to the antimicrobial agent (Kwang and Abbott 2014; Love and Jenkinson 2002).

AH Plus demonstrated a 5‐log decrease in values compared to controls in the CFU analysis. Conversely, AH Plus was able to reduce the DNA units by only 1.5‐log. There were no appreciable variations between the evaluated materials using both methodologies (culture and qPCR). A similar trend was observed in alpha diversity analysis. The next‐generation sequencing analysis indicated that AH Plus was the only material that impacted the biofilm community composition (ANOSIM *R* = 0.674, *p* < 0.001). Studies conducted by Leonardo et al. (2000) and Siqueira and de Uzeda (1996) revealed that AH Plus demonstrated some antibacterial properties, exhibiting an ability to inhibit bacterial growth. In vitro research by Saleh et al. (2004) who used a similar infection model, indicated that AH Plus was effective in eradicating *E. faecalis* within dentinal tubules. Saavedra et al. (2022) also found that AH Plus altered not only the community composition but also inhibited biofilm formation. The antibacterial effect of epoxy resin‐based materials might be related to the bisphenol‐F, dibenzyldiamine component, or the presence of unreacted monomers post‐polymerisation (Saavedra et al. 2022). This effect has also been observed previously using single species biofilms (Zhang et al. 2009; Kapralos et al. 2018). This finding is critical, as it could suggest that AH Plus could impact the organisation of the biofilm community rather than just reducing total bacterial load.

A limitation of this study is the complexity of interpreting changes in microbial communities. Biofilms are dynamic ecosystems, and shifts in specific taxa—such as *Fusobacterium* or *Prevotella*—can be balanced out by other species taking on similar functions, a concept known as functional redundancy (Ordinola‐Zapata et al. 2024). Therefore, the full clinical impact of community changes is unknown. The determination of residual bacterial localization is another limitation of this study and is a drawback of all studies that use culture or molecular techniques. CLSM can identify the residual biofilm but is unable to identify the bacterial taxa or contaminants (Zapata et al. 2009; Ordinola‐Zapata et al. 2012). Other limitations of the CLSM technique are the potential of autofluorescence produced by dentin or residual sealer, which can lead to false positives, or limited laser penetration. It is also important to mention that next‐generation sequencing generates ‘big data’ because of the high dimensionality; some indices like the number of OTUs and the Chao 1 can present high standard deviations because of the inconsistent presence of multiple low abundant oral taxa among samples. For this study, those taxa represented less than 1.5% of the bacteria identified. The Shannon index combines the biofilm richness and evenness into a single digit, producing values that are more stable. For better comparison of community composition, beta diversity is an accepted analysis that does not rely on F‐statistics. This analysis can reveal the community differences among different groups (Alquria et al. 2024).

Although we recognise that laboratory experiments cannot fully replicate the complexities of the clinical setting in which multiple variables can affect bacterial survival, in vitro studies remain relevant to understanding the biomaterial‐biofilm interaction without the presence of other confounding factors (Wang et al. 2012; Kreth et al. 2019). The findings of this study emphasise the importance of evaluating the antimicrobial efficacy of new root canal sealers and intracanal medicaments not only in terms of bacterial reduction but also in their ability to impact the biofilm community on infected dentine, which may have effects on microbial persistance.

## 5 Conclusion

Calcium hydroxide and BC Sealer significantly reduced the viability and the total number of DNA copies. AH Plus sealer reduced the bacterial viability but did not affect the DNA concentration. AH Plus significantly alters overall biofilm community composition compared to other groups. None of the materials tested eliminated the multispecies biofilm completely.

## Author Contributions

**Steven K. Uttech:** writing and data acquisition. **Ronald Ordinola‐Zapata:** conceptualization, methodology, data acquisition and writing. **W. Craig Noblett:** review and editing. **Maria Martell:** software and bioinformatic analysis. **Bruno Lima:** review and editing. **Christopher Staley:** writing, software and bioinformatic analysis.

## Ethics Statement

This in vitro experiment was not considered human research (Protocol 00024340, 14237).

## Conflicts of Interest

The authors declare no conflicts of interest.

## Supporting information


**Figure S1:** PRILE flowchart.

**Figure S2**illustration showing the main procedures performed during the experimen. Sample analysis and biofilm reactor illustrations were generated using chatgpt. 

## Data Availability

The data that support the findings of this study are available from the corresponding author upon reasonable request.
