# Supplementary material for: Multispecies Biofilms Treated With Endodontic Sealers or Calcium Hydroxide: Antimicrobial Activity and Changes in Community Composition
Source: Int Endod J. 2025 Aug 13;58(11):1764–73. doi: 10.1111/iej.70015 (PMC12518701; doi:10.1111/iej.70015)
Supplement: Supplementary file 1 — Figure S1: PRILE flowchart. [file IEJ-58-1764-s001.docx]

**PRILE 2021 Flowchart**
