# Supplementary material for: Multispecies Biofilms Treated With Endodontic Sealers or Calcium Hydroxide: Antimicrobial Activity and Changes in Community Composition
Source: Int Endod J. 2025 Aug 13;58(11):1764–73. doi: 10.1111/iej.70015 (PMC12518701; doi:10.1111/iej.70015)
Supplement: Supplementary file 2 — Figure S2illustration showing the main procedures performed during the experimen. Sample analysis and biofilm reactor illustrations were generated using chatgpt. [file IEJ-58-1764-s002.pdf]

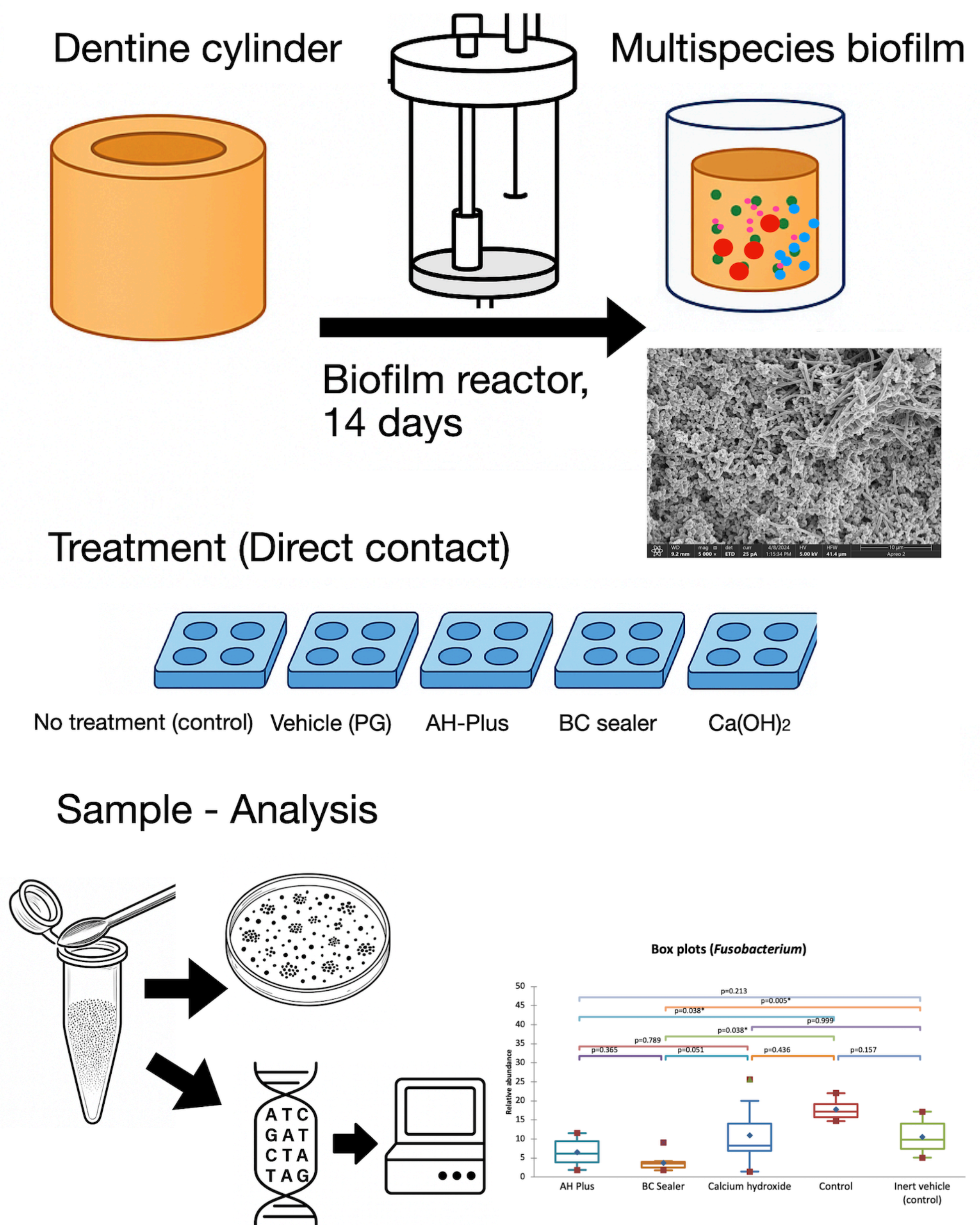
Dentine cylinder
Multispecies biofilm
Biofilm reactor,
14 days
Treatment (Direct contact)
No treatment (control)
Vehicle (PG)
AH-Plus
BC sealer
Ca(OH)2
Sample - Analysis
A T C
G A T
C T A
T A G
Box plots (Fusobacterium)
Relative abundance
50
45
40
35
30
25
20
15
10
5
0
p=0.213
p=0.005*
p=0.038*
p=0.999
p=0.038*
p=0.789
p=0.365
p=0.051
p=0.436
p=0.157
AH Plus
BC Sealer
Calcium hydroxide
Control
Inert vehicle
(control)
